# Effect of Earthing Mats on Sleep Quality in Rats

**DOI:** 10.3390/ijms25189791

**Published:** 2024-09-10

**Authors:** Minsook Ye, Woojin Jeong, Hyo-jeong Yu, Kyu-ri Kim, Sung Ja Rhie, Yongsuk Kim, Jiyoun Kim, Insop Shim

**Affiliations:** 1 Department of Physiology, College of Medicine, Kyung Hee University, Seoul 02453, Republic of Korea; jh486ms22@naver.com (M.Y.); tnftlfgwl@naver.com (W.J.); yyllzzll@naver.com (H.-j.Y.); kyuri_kim@khu.ac.kr (K.-r.K.); 2 Department of Beauty Design, Halla University, Wonju 26404, Republic of Korea; sjlee@halla.ac.kr; 3 DF World Corporation, Royal Building, 19 Saemunan-ro 5-gil, Jongno-gu, Seoul 03173, Republic of Korea; kimyongsuk0722@worldhomedr.com; 4 World Home Doctor Corporation, 73 Anyangcheonseo-ro, Manan-gu, Anyang-si 14087, Republic of Korea; vejeun@worldhomedr.com

**Keywords:** earthing mat, electroencephalography (EEG), sleep disturbances, orexin, SOD1

## Abstract

Grounding, a therapeutic technique involving direct contact with the earth, has been proposed by various studies to potentially have beneficial effects on pressure, sleep quality, stress, inflammation, and mood. However, the scientific evidence supporting its sedative effects remains incomplete. This study examined the sedative effectiveness of an earthing mat on sleep quality and investigated the underlying neural mechanisms using electroencephalography (EEG) analysis in rodents, focusing on orexin and superoxide dismutase (SOD) levels in the brain. Rats were randomly assigned to four groups: the naïve normal group (Nor), the group exposed to an earthing mat for 7 days (A-7D), the group exposed to an earthing mat for 21 days (A-21D), and the group exposed to an electronic blanket for 21 days (EM). EEG results revealed that the A-21D group exhibited significantly reduced wake time and increased rapid eye movement (REM), non-rapid eye movement (NREM), and total sleep time compared to the Nor group (*p* < 0.05). Moreover, the A-21D group demonstrated a significant increase in NREM sleep (*p* < 0.001), REM sleep (*p* < 0.01), and total sleep time (*p* < 0.001), along with a decrease in wake time compared to the EM group (*p* < 0.001). The orexin level in the A-21D group was significantly lower compared to the Nor group (*p* < 0.01), while SOD1 expression was markedly elevated in the A-21D group compared to the Nor group (*p* < 0.001). These results suggest that the earthing mat may represent a promising new method for promoting sleep quality and could serve as an effective therapeutic technique.

## 1. Introduction

Sleep is a state of rest for the mind and body, characterized by reduced sensory responsiveness to external stimuli and altered consciousness, distinct from wakefulness [1]. It plays an essential role in maintaining overall health by facilitating the restoration of normal functions across various body systems. Sleep is particularly critical for regulating mood, enhancing cognitive performance, and restoring the functions of the endocrine and immune systems [2]. Traditionally, pharmacological treatments such as benzodiazepines and barbiturates have been employed to manage sleep disorders or insomnia [3]. However, these medications are associated with potential adverse effects, including dependency, cognitive impairment, and residual daytime sedation [4]. To mitigate these risks, ongoing research is increasingly exploring non-pharmacological interventions.

Among non-pharmacological interventions, earthing, also known as grounding, refers to the practice of direct physical contact between the body and the ground, which may have various health implications [5]. Earthing has been linked to pain reduction, modulation of immune responses, and decreased levels of inflammation-related biomarkers, indicating potential benefits for inflammatory and autoimmune conditions [6]. Numerous studies have reported that earthing can exert neuromodulatory effects on the brain, influencing nervous system dysfunction [7]. A randomized controlled trial has demonstrated that earthing can enhance not only pain management but also mood, fatigue, overall health, and quality of life. Despite extensive research, a definitive mechanism underlying the preclinical treatment or improvement of sleep and related disorders through earthing remains to be fully elucidated.

Orexin neurons in the lateral hypothalamus (LH) are crucial for promoting and sustaining arousal. The orexin system is recognized as a significant target in the regulation of endogenous adenosine-mediated sleep homeostasis. Orexins, neuropeptides expressed exclusively by neurons in the LH, are implicated in a variety of functions, including feeding, addiction, and the regulation of sleep/wake cycles [8,9]. The critical role of the orexin system in sleep/wake cycle regulation has been underscored by both genetic and pharmacological studies. Research indicates that mice lacking orexin exhibit narcoleptic-like symptoms, and postmortem analyses of brain tissue from narcolepsy patients have shown a near-complete loss of orexin neurons, thereby providing substantial evidence of the importance of orexins in humans [10]. Numerous OX1 and OX2 receptor antagonists have demonstrated sleep-promoting effects in both animal models and clinical studies. These antagonists have been shown to induce physiological sleep architecture in humans, characterized by increased durations of both rapid eye movement (REM) and non-rapid eye movement (NREM) sleep. Furthermore, these antagonists increase REM and NREM sleep in mice. Consequently, the blockade of orexin receptors represents a novel approach for the treatment of insomnia.

Inadequate sleep results in diminished energy and vitality, which can lead to various health issues. During sleep, numerous physiological processes are activated, providing the body with an opportunity to rest and recover. Most organ systems remain in a resting state during sleep, contributing to the restoration of normal physiological functions [1]. Some of these functions are associated with antioxidant defense mechanisms. Prior research indicates that reactive oxygen species (ROS) accumulated during wakefulness are removed during sleep [11]. It has been proposed that the accumulation of oxidative substances decreases during sleep, leading to enhanced efficiency of antioxidant mechanisms. Consequently, superoxide dismutase (SOD) may contribute to reducing oxidative stress and protecting cells by decreasing the accumulation of oxidative substances and facilitating the breakdown of free radicals during sleep [12]. Several studies have demonstrated that sleep deprivation impairs the ability to eliminate oxidative stress in the brains of rats and mice [12,13].

The aim of the present study was to investigate the effects of an earthing mat on the sleep–wakefulness cycle and its potential to enhance sleep induced by pentobarbital, using electroencephalographic (EEG) recordings in an animal sleep model. Additionally, the study sought to examine the impact of the earthing mat on the levels of SOD and orexin in the LH. We anticipated that this study would elucidate the effects of the earthing mat on sleep and provide insights into its influence on sleep-related mechanisms, thereby laying the groundwork for future research on the interactions between earthing and sleep regulation.

## 2. Results

### 2.1. Effect of Earthing Mat on EEG Sleep Architecture and Profile

**Figure 1:**
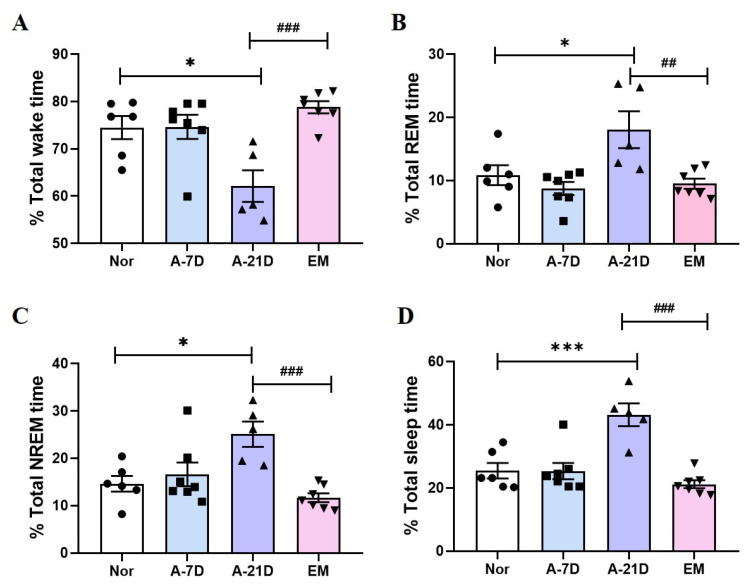
Effect of earthing mat on sleep architecture. Changes in the percentage of wake time (**A**), REM sleep (**B**), NREM sleep (**C**), and total sleep (**D**) during the dark phase are depicted in the earthing mat-exposed groups. The data represent the mean ± SEM of the percentage of time spent in the sleep–wake state. *** *p* < 0.001, * *p* < 0.05 vs. Nor, ### *p* < 0.001, ## *p* < 0.01 vs. A21-D; one-way ANOVA followed by Tukey. ● Nor, ■ A-7D,▲ A-21D,▼EM.

The effect of the earthing mat on EEG sleep architecture and profile is shown in Figure 1. EEG signals were recorded for 12 h. In the A-21D group, there was a significant reduction in wake time (*p* < 0.05, Figure 1A) and a marked increase in REM (*p*  <  0.05, Figure 1B), NREM (*p*  <  0.05, Figure 1C), and total sleep (*p*  <  0.001, Figure 1D) compared with the Nor group. The A-21D group exhibited a significantly decreased wake time (*p*  <  0.001, Figure 1A) and increased REM (*p*  <  0.01, Figure 1B), NREM (*p*  <  0.001, Figure 1C), and total sleep (*p*  <  0.001, Figure 1D) compared to the EM group.

### 2.2. Effect of Earthing Mat on the Number of Orexin Neurons in the LH

**Figure 2:**
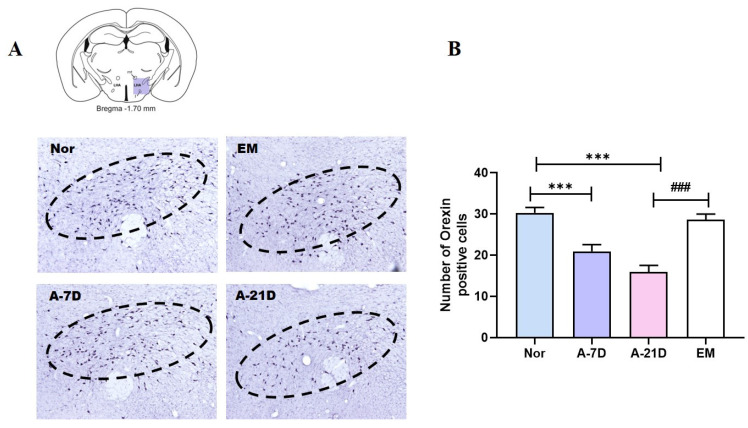
Impact of earthing mat on orexin-positive cells in the LH. (**A**) Photomicrographs illustrating orexin-positive cells in the LH. The dashed circles indicate the LH region. (**B**) Quantification of orexin-positive cells in the LH. *** *p* < 0.01 vs. Nor; ### *p* < 0.05 vs. A-21D; one-way ANOVA followed by Tukey’s test.

Orexin neurons within the LH are pivotal in governing the modulation of sleep–wake patterns. We examined orexin expression in the LH (Figure 2) and found that the number of orexin-positive cells was lower in the A-7D group and A-21D group than in the Nor group (*p*< 0.001; Figure 2). The A-21D group exhibited a significantly decreased number of orexin-positive cells compared to the EM group (*p*  <  0.001, Figure 2).

### 2.3. Effect of Earthing Mat on the Number of SOD-Positive Cells in the LH

**Figure 3:**
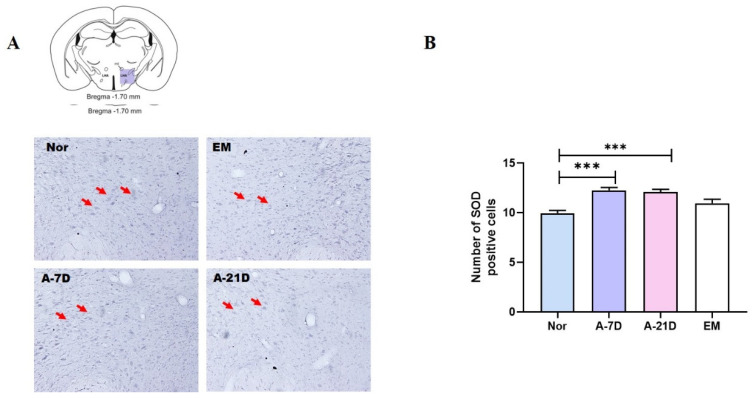
Impact of earthing mat on SOD-positive cells in the LH. (**A**) Photomicrographs illustrating orexin-positive cells in the LH. (**B**) Quantification of SOD-positive cells in the LH. *** *p* < 0.01 vs. Nor; one-way ANOVA followed by Tukey’s test.

The evaluation of the SOD-immunoreactive cells per section of the LH is shown in Figure 3. The expression of the SOD-immunoreactive neurons in the A-7D and A-21D groups was significantly increased compared to the Nor group (*p*  <  0.001, Figure 3).

## 3. Discussion

The global rise in sleep disorders has emerged as a significant health issue, affecting not only individuals’ physical health but also their mental well-being and overall quality of life. The complex neurobiological mechanisms underlying these disorders are the subject of ongoing research, aiming to elucidate the intricacies of sleep regulation and identify effective treatments for sleep-related conditions. Non-pharmacological treatments are increasingly recognized for their potential benefits, leveraging both traditional practices and contemporary scientific insights. Notably, earthing has garnered attention for its broad spectrum of potential therapeutic effects. Clinical studies have demonstrated that earthing can enhance sedation and sleep quality, as well as alleviate anxiety and provide antidepressant and anti-inflammatory benefits [6,14]. These findings are critical for developing comprehensive treatment strategies that address both physiological and psychological aspects of sleep disorders, potentially offering safer and more natural alternatives to conventional pharmacological treatments.

The current study provides experimental evidence supporting the sedative effects of earthing mat exposure in an animal model. Exposure to the earthing mat resulted in a significant reduction in total wake time and an increase in both REM and NREM sleep durations. Additionally, orexin expression in the lateral hypothalamus (LH) was reduced after 21 days of exposure, while SOD levels in the LH were elevated after both 7 and 21 days of exposure. These results suggest that earthing mat exposure not only has sedative effects but also enhances sleep by activating sleep-promoting regions in the brain, indicating its potential as a novel non-pharmacological therapeutic approach.

Previous research has shown that earthing promotes the transition of the brain to alpha wave activity and enhances overall sleep quality [15]. Alpha waves, associated with relaxation and mental calmness, play a critical role during REM sleep and deep restorative sleep [16]. Therefore, the enhancement of alpha wave activity through earthing may lead to improvements in REM sleep and contribute to overall sleep recovery. Consistent with these findings, the present study demonstrates that exposure to an earthing mat reduces wake time and significantly enhances both REM and NREM sleep.

The LH is a critical brain region involved in regulating the sleep–wake cycle. Orexin neurons in the LH are known for their wakefulness-promoting activity, exhibiting peak firing rates and the highest extracellular levels of orexin during wakefulness [17]. The LH coordinates sleep-promoting activities, with previous studies indicating that orexin helps maintain a balance between sleep and wakefulness by inhibiting NREM and REM sleep while promoting wakefulness to a lesser extent [18]. Orexin-deficient animal models have shown increased REM sleep, underscoring the importance of investigating the roles of the LH and orexin in understanding and addressing sleep-related disorders [19]. In this study, exposure to the earthing mat was found to decrease the expression of orexin-positive cells in the LH region. Furthermore, A-21D may reduce orexin levels in the LH in a dose-dependent manner, potentially leading to a reduction in arousal and an enhancement in sleep quality.

During sleep, the brain undergoes recovery and regeneration processes that contribute to a reduction in oxidative stress. Research indicates that sleep deprivation can decrease the expression of SOD, an important antioxidant enzyme associated with oxidative damage in the brain. Conversely, adequate sleep helps maintain SOD activity and facilitates the removal of reactive oxygen species (ROS). Previous studies have shown that antioxidant enzyme activity, including SOD, increases in animal models subjected to sleep deprivation. Consistent with these findings, the present study demonstrates that exposure to A-7D and A-21D significantly increased SOD levels in the LH. This result suggests that these treatments may reduce oxidative stress and improve the brain’s oxidative defense system. The observed increase in SOD levels supports the hypothesis that interventions designed to maintain or restore antioxidant enzyme activity can mitigate oxidative damage associated with sleep deprivation and enhance overall brain health.

This study presents a potential non-pharmacological therapeutic option for human sleep disorders. Utilizing parameters such as EEG, orexin, and SOD, we investigated the impact of the earthing mat on sleep in a rat model. The results indicated that the effects of the earthing mat became more pronounced with longer exposure, leading to a significant reduction in wakefulness and an increase in total sleep duration. Additionally, levels of orexin in the LH decreased, while SOD levels increased. These findings suggest that the effects of the earthing mat may be attributed to its antioxidant properties and its impact on the sleep-promoting center, LH. Consequently, the earthing mat could represent a valuable alternative to pharmacological sleep aids. However, the specific mechanisms by which the earthing mat influences wakefulness, REM, and NREM sleep have not yet been fully elucidated. Further research is necessary to clarify the precise neural mechanisms involved in sleep promotion. This study has several limitations. Firstly, the small sample size employed may restrict the generalizability of the results. Secondly, the findings derived from animal models may not fully translate to human physiological responses, underscoring the need for additional studies involving human subjects to confirm these outcomes. Addressing these limitations in future research is essential to improve the reliability and applicability of the findings.

## 4. Materials and Methods

### 4.1. Animals and Grouping

Adult male Sprague Dawley rats were obtained from Samtako (Osansi, Gyeonggi-do, Republic of Korea). These animals were accommodated in a climate-controlled environment with temperatures maintained between 20 ° and 25 °C and humidity levels set at 45% to 65%, following a 12-h light and 12-h dark cycle (lights on at 8 a.m.). Food and water were provided ad libitum throughout the study. The laboratory animals were handled and treated in compliance with the guidelines outlined by the Ministry of Food and Drug Safety (MFDS) National Institute of Toxicological Research, per the standards for laboratory animal care and usage (Approval No. KHUASP(SE)-14-051).

### 4.2. Earthing Mat

**Figure 4:**
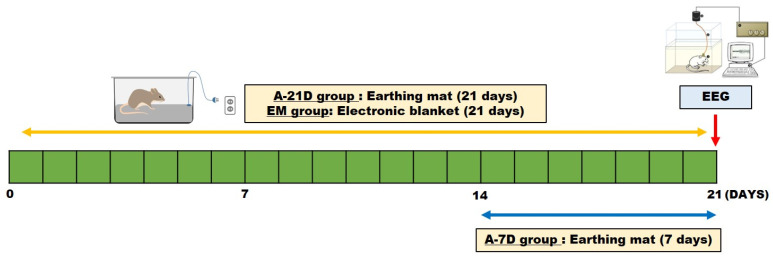
Animal groups and treatments in the experimental design of this study.

The earthing mat utilized in this study was supplied by the World Home Dr. Company (Anyang City, Kyunggido, Republic of Korea). The earthing mat system comprises a cotton sheet, and the electric emission plate is linked to a ground port of an electrical outlet, as illustrated in Figure 4. The grounding port facilitates the reconnection of the rats’ conductive bodies to the Earth’s natural and subtle surface electric charge. Rats were positioned on the earthing mat preceding behavioral tests.

### 4.3. EEG Surgery

The subjects were divided into four groups: normal (Nor; *n* = 6), earthing mat exposure for 7 days (A-7D; *n* = 7), earthing mat exposure for 21 days (A-21D; *n* = 5), and electronic blanket exposure for 21 days (EM; *n* = 7). Electroencephalogram (EEG) electrodes were surgically implanted to facilitate polygraphic recordings, following the guidelines delineated in the Paxinos and Watson stereotaxic atlas [20]. Surgical anesthesia was induced with intraperitoneal pentobarbital (40 mg/kg). Subsequently, the rats were subjected to chronic implantation of a head mount. The transmitter body was subcutaneously placed off the midline, posterior to the scapula, secured to the skin, and stabilized using three sutures. Skull-mounted electrodes were secured with screws and dental cement. All surgical interventions were performed using a stereotaxic methodology in an aseptic environment. Postoperatively, each rat was allowed a 7-day recovery period in separate transparent enclosures.

### 4.4. Methodology of EEG Recording

After recovery, the rats were habituated to the recording conditions before the test. The rats were recorded at baseline and after 21 days of exposure to the earthing mat, prior to EEG recording. Recording began at 6:00 p.m., and 12 h of EEG and activity were recorded in all rats. Cortical EEG signals were amplified (×100), filtered (low-pass filter; 100 Hz EEG), digitized at a sampling rate of 100 Hz, and recorded with the PAL-8200 data acquisition system (Pinnacle Technology Inc., Lawrence, KS, USA) using a chart speed of 25 mm/s. SleepSign Ver. 3 software (Kissei Comtec, Nagano, Japan) automatically classified sleep–wake states into three categories: wakefulness (Wake), rapid eye movement (REM) sleep, and non-REM (NREM) sleep.

### 4.5. Staining of the LH

After transcranial perfusion, the rat brains were removed with 4% formaldehyde solution (Sigma-Aldrich Co. St. Louis, Missouri, USA), post-fixed in the same fixative for 24 h, and placed in phosphate-buffered saline containing 20% sucrose for 72 h. Serial 30 µm thick coronal sections were cut using a cryostat microtome (CM1850UV; Leica Microsystems Inc., Wetzlar, Germany) and were stored at −20 °C; they were histochemically processed as free-floating sections. Sections were washed three times with PBST.

Primary rabbit polyclonal antibodies against orexin (Abcam, Cambridge, MA, USA) and SOD1 (Abcam, Cambridge, MA, USA) were diluted to 1:800. The sections underwent a 12-h incubation at 4 °C with constant agitation. After rinsing with PBST, a 2-h incubation at room temperature was performed using a biotinylated goat anti-rabbit antibody (Vector Laboratories, Inc., Burlingame, CA, USA) diluted to 1:200 in PBST with 2% v/v normal goat serum. Subsequently, the sections were exposed to an avidin–biotin–peroxidase complex reagent (Vector Laboratories) for 2 h at room temperature. After further rinsing with PBST, the tissues were developed using a DAB substrate kit (Vector Laboratories). The final steps included mounting the sections on slides, air-drying, and covering them for microscopic observation.

### 4.6. Data Analysis

All statistical analyses were conducted using SPSS (IBM^Ⓡ^ SPSS^Ⓡ^ Statistics Ver. 23 Chicago, IL, USA). For multiple comparisons, behavioral data were analyzed using one-way analysis of variance (ANOVA). Tukey’s post hoc test was used to identify significant differences among groups. The level of significance was set at *p* < 0.05.

## Data Availability

The data used to support the findings of this study are available from the corresponding author upon request.
